# Orchids of Azerbaijani Cemeteries

**DOI:** 10.3390/plants10122779

**Published:** 2021-12-16

**Authors:** Attila Molnár V., Viktor Löki, Marc Verbeeck, Kristóf Süveges

**Affiliations:** 1 Department of Botany, University of Debrecen, Egyetem tér 1, H-4032 Debrecen, Hungary; eska1994@gmail.com; 2 Wetland Ecology Research Group, Centre for Ecological Research, Bem tér 18/C, H-4026 Debrecen, Hungary; loki.viktor@ecolres.hu; 3 Arbeitskreis Heimische Orchideen Baden-Württemberg, D-69469 Weinheim, Belgium; mv2know@hotmail.com

**Keywords:** anthropogenic habitats, Caucasus, *Himantoglossum formosum*, human-made habitats, Orchidaceae, salep harvesting, Transcaucasia

## Abstract

In order to explore their orchid flora, we performed surveys of 96 Azerbaijani burial places in 2018 and 2019. Altogether, 28 orchid taxa were found in 37 visited cemeteries. In the orchid diversity a remarkable pattern was observed: geographic latitude was significantly and positively related to the number of taxa and number of individuals. The most widespread and abundant orchids in Azerbaijani graveyards were *Anacamptis pyramidalis* and *A. papilionacea* (found in 23 and 8 cemeteries, respectively). Azerbaijani cemeteries can be important refuges for rare and threatened orchids, e.g., *Himantoglossum formosum* (three cemeteries), *Ophrys sphegodes* subsp. *mammosa* (eight), *Orchis adenocheila* (two), *O. punctulata* (three), *O. stevenii* (one) and *Steveniella satyrioides* (one). *Epipactis turcica*, detected in a single locality, was previously unknown to the flora of Azerbaijan. Additionally, we documented orchid tuber (salep) collection in two cemeteries.

## 1. Introduction

The Earth’s surface has changed dramatically in recent centuries, with human activities serve as a leading cause of the drastic reduction in the area of natural habitats [1,2]. In parallel with the degradation and fragmentation of natural environments throughout the world, isolated natural habitat patches as remnants of the original wildlife have been revalued [3]. Anthropogenically influenced habitats now occupy a significant part of the Earth’s surface and expand rapidly [4]. In order to conserve the remaining biodiversity, it is of the utmost importance to identify and protect the remaining habitats with a high conservation value, to develop a sustainable habitat management practice, and to plan future developments in the light of nature conservation priorities [5].

Recently, conservation professionals have recognized that some of the anthropogenically influenced or even human-made habitats, such as abandoned mines and industrial sites [6,7,8], road verges [9,10,11], tree plantations [12,13,14], river dikes [15], burial mounds [16], and urban habitats [17,18], play significant roles in conserving biodiversity. During the last decades, it has become increasingly evident that cemeteries also play an important role in maintaining biodiversity [19]. Although the orchid flora of cemeteries is globally rather poorly known, occurrences of orchids were published from Australian, Asian, and European burial places [20]. Based on previous knowledge on the occurrence and diversity of orchids in Turkish [21,22,23,24], Albanian [25] and central European [26] burial grounds, we predicted potential conservational importance of traditional Caucasian cemeteries. One of the main goals of our study was to search for *Himantoglossum formosum*, the rarest and perhaps the least known orchid of the Caucasian region [27]. During the 180 years after its description [28], almost nothing was known about the species [29], and it was re-discovered in 1994 [30]. According to recent studies, this is an ancient, phylogenetically isolated [31] and morphologically well separated [32] bona fide species. It is listed as Vulnerable (Rare) in the IUCN Red List of Threatened Plants [33].

The aims of this paper were to survey Azerbaijani cemeteries as orchid habitats, and to test which geographic factors influence the prevalence of orchids in the surveyed cemeteries.

## 2. Materials and Methods

**Figure 1:**
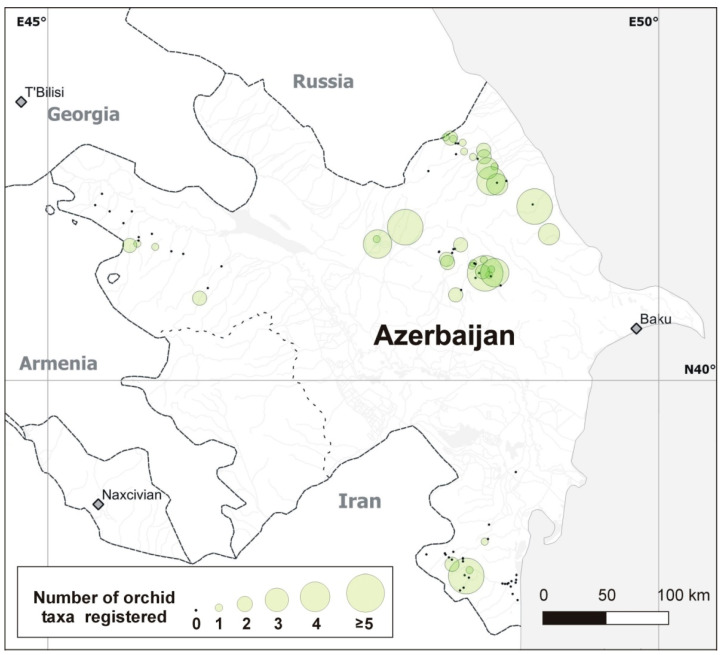
Number of orchid taxa in the cemeteries surveyed.

We studied burial grounds (Azerbaijani: məzarlıq, hereafter cemeteries) regardless of their spatial dimension, position within settlements, or presence of built facilities. We surveyed 96 Azerbaijani cemeteries (Figure 1, Table A1) during 2018 (17–30 May by Molnár V., Löki, Mizsei and Süveges, and 28 June–4 July by Molnár V. and Szabó) and 2019 (29 April–6 May by Verbeeck, Duijnhouwer, Segers and Bobocea) and (31 May–6 June by Verbeeck, Duijnhouwer and Bradeanu). Most cemeteries were visited only once (90 and 3 cemeteries in May 2018 and in April 2019, respectively), but three cemeteries were visited in both years. All orchid taxa and the number of individuals were counted or estimated in the whole area of each visited cemetery. Species were identified based on the comprehensive book of Kuehn et al. [34]. Authors of plant names were listed in Table 1. The geocoordinates and the elevation of the visited cemeteries were determined using a Garmin eTrex Legend handheld GPS device and recorded in WGS84 format. During field trips, particular attention was devoted to documenting salep collection activity in cemeteries.

To understand the role of geographic factors in determining variation in taxon richness and abundance of orchids across Azerbaijan, we built statistical models with either of these variables as dependent variables, and latitude, longitude and altitude as explanatory variables. Both the number of individuals and the number of taxa had Poisson distributions, but due to the overdispersion in these variables, we used generalized linear model (GLMs) with quasi-Poisson distribution. All models were built in the R statistical environment [35].

## 3. Results

Numbering (ID), geographic location, and altitude above see level of the cemeteries visited, together with lists of the orchid taxa found in each one, are given in Table A1. In total, 28 orchid taxa were found, and considerable differences can be observed in the number of individuals and frequency of each taxon (Table 1), as well as in orchid species richness and abundance of each cemetery (Table 2).

**Figure 2:**
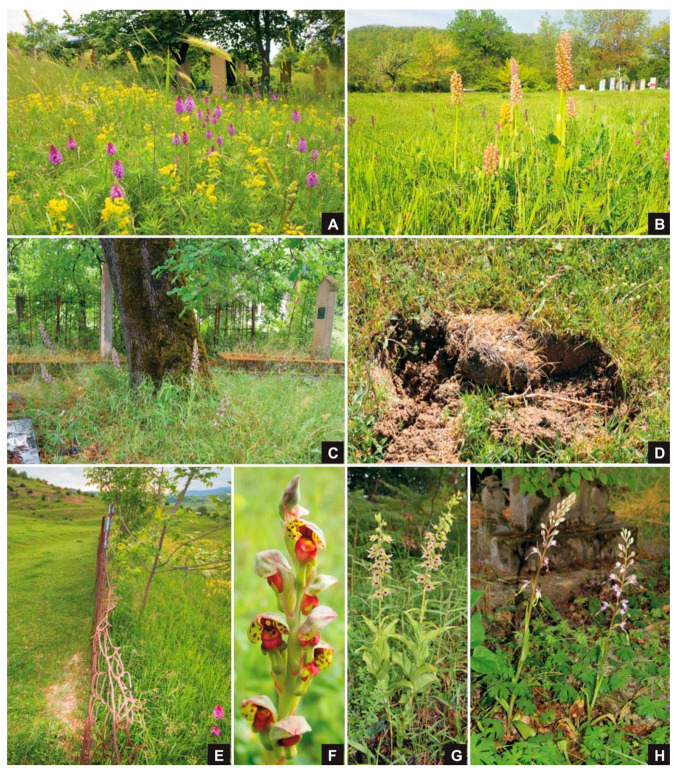
Orchids in Azerbaijani cemeteries. (**A**) *Anacamptis pyramidalis* population in the cemetery of Əngixaran (AZ–61). (**B**) Cemetery of Nohurqishlaq (AZ-93), habitat of *Orchis punctulata*, *O. stevenii* and their hybrids (*Orchis ×chabalensis*). (**C**) Viable population of *Himantoglossum formosum* was found on a few tens of square meter of refuge under some old oak trees in cemetery of Zizik (AZ-74). (**D**) Spurs of salep harvesting in the cemetery of Ağabəyli (AZ-52). (**E**) Effect of fencing around cemetery against grazing: plant cover is considerable lower outside (left) than inside (right, with flowering individuals of *Anacamptis pyramidalis*) of cemetery of Zurnabad (AZ-32). (**F**) Inflorescence of *Steveniella satyrioides*. (**G**) Occurrence of *Epipactis turcica* was formerly unknown from Azerbaijan (Tengealti, AZ-85). (**H**) A very localized and rare endemic species, *Himantoglossum formosum* in cemetery of Zizik (AZ-74). Photo credit: **A**, **C**, **D**, **G** and **H** by A. Molnár V.; **B** and **F** by M. Verbeeck; **E** by V. Löki.

Each taxon was found total in 1–24 cemeteries (mean ± SD = 3.2 ± 4.5), with the number of individuals varying from 1 to 1902 (mean ± SD = 150 ± 374). The most widespread and abundant species was *Anacamptis pyramidalis* (Figure 2A). The number of taxa detected in only one graveyard was 15, whereas four species were found in more than five cemeteries. The highest number of taxa in a given cemetery was 9. In most cases only one taxon (18 cemeteries (15%)) or two taxa (11 cemeteries (9.4%)) occurred. Cemeteries that serve as habitats for five or more taxa were extremely rare (4 (3.4%)). The most orchid-rich cemeteries were found near Lerik (AZ-16, 9 species) Ağabəyli (AZ-52, 8 species), Nohurqishlaq (AZ-93, Figure 2B, 8 species), and DashliJalgan (AZ-90, 5 species). 

The harvest of orchid tubers (“salep”) was observed in two cemeteries during 2018. In Ağabəyli cemetery (AZ-52, Figure 2D) three species (*Anacamptis papilionacea, Orchis adenocheila, O. simia*), and in Dashli Jalgan cemetery (AZ-90) five species (*Anacamptis collina, A. papilionacea, Ophrys sphegodes* subsp. *mammosa, Orchis simia, Neotinea tridentata*), were collected. Both of these localities host notable orchid populations with eight and five species, respectively.

The number of orchid taxa and individuals found in Azerbaijani cemeteries was significantly positively related to latitude (Table 3 and Table 4, respectively), but not to longitude and altitude. When non-significant predictors were removed from the model in a stepwise manner (based on the largest *p*-values), only latitude remained in the final model as a significant predictor of orchid species richness and abundance.

## 4. Discussion

During our work, it has been proved that Muslim Azerbaijani cemeteries host significant orchid populations. The key conservation importance of Azerbaijani cemeteries can be explained by two facts: (1) Religious privileges protected these sacred sites and their natural values, because they have largely been exempt from forest and agricultural utilization ever since; and (2) the mostly fenced area of cemeteries provide protection against excessive grazing (Figure 2E). 

Azerbaijani cemeteries provide shelters for several valuable populations of rare and threatened orchids. From a conservation point of view, one of the most valuable species is the Eastern Caucasian endemic *Himantoglossum formosum* (Figure 2H), which was found in three of the visited cemeteries (Zizik, AZ-74, Figure 2C; Yasab, AZ-78; Piral, AZ-79). Viable populations of the rare *Orchis adenocheila* were found in two cemeteries (Lerik, AZ-16; Ağabəyli, AZ-52). The occurrence of *Steveniella satyrioides* was detected in cemetery of Lerik (Lerik, AZ-16, Figure 2F). The occurrence of *Epipactis turcica* (Figure 2G) was also found near Tengealti (AZ-85); this taxon was formerly unreported in Azerbaijan.

The long-term survival of these orchid populations in cemeteries strongly depends on long-established, sustainable management practices and traditional burial habits [22,36]. Establishment of graves (especially modern graves covered by marble or concrete tombstones) on the most valuable parts of these cemeteries is expressly undesirable from a conservation perspective, as well as the use of herbicides or electric trimmers. However, mowing or moderate grazing of grassy areas around the burial ground is preferred and encouraged for a more efficient conservation of the local biodiversity and valuable flora elements. Based on their diverse and abundant orchid community in some of the visited cemeteries, we strongly recommend the local councils and the nature protection authorities to protect certain burial places, especially near Lerik (AZ-16), Ağabəyli (AZ-52), DashliJalgan (AZ-90), Nohurqishlaq (AZ-93), and Nugadi (AZ-92). 

A special threatening factor of tuberous orchids, namely the harvest of their tubers (making salep for culinary purposes [37]) was observed in Azerbaijani cemeteries. On the one hand, the right of local human communities to continue using traditional natural resources is unquestionable and seems also sustainable [38,39]. On the other hand, the effects of tuber collection on populations of frequent and widespread orchids is little known, while the sustainability of salep harvesting is at least controversial [40,41,42,43,44,45,46,47]. However, destroying the rarest taxa (*Himantoglossum formosum, Orchis adenocheila*) should definitely be avoided.

## Figures and Tables

**Table 1:** Orchid taxa recorded in Azerbaijani cemeteries.

Taxon	No. of Cemeteries	Total Number of Individuals
*Anacamptis pyramidalis* (L.) Rich.	23	1901
*Anacamptis papilionacea* L.	8	567
*Ophrys sphegodes* subsp. *mammosa* (Desf.) Soó ex Nelson	8	227
*Orchis simia* Lam.	7	492
*Limodorum abortivum* (L.) Sw.	4	23
*Anacamptis morio* (L.) Bateman et al.	3	514
*Anacamptis collina* (Banks and Sol. ex Russell) Bateman et al.	3	86
*Himantoglossum formosum* (Steven) K. Koch	3	27
*Orchis punctulata* Steven ex Lindl.	3	14
*Cephalanthera rubra* (L.) Rich.	3	9
*Ophrys oestrifera* M. Bieb.	3	3
*Orchis adenocheila* Czerniak.	2	239
*Orchis mascula* subsp. *longicalcarata* Akhalk. et al.	2	9
*Anacamptis coriophora* (L.) Bateman et al.	2	6
*Ophrys apifera* Huds.	2	3
*Orchis caucasica* Regel	1	50
*Orchis ×chabalensis* B. Baumann et al. (*O. punctulata × O. stevenii*)	1	30
*Ophrys* sp.	1	24
*Dactylorhiza romana* (Sebast.) Soó	1	20
*Orchis stevenii* Rchb. F.	1	20
*Epipactis turcica* Kreutz	1	7
*Anacamptis* sp.	1	6
*Ophrys caucasica* Woronow ex Grossh.	1	5
*Epipactis microphylla* (Ehrh.) Sw.	1	3
*Neotinea tridentata* (Scop.) Bateman et al.	1	3
*Epipactis* sp.	1	2
*Cephalanthera damasonium* (Mill.) Druce	1	1
*Steveniella satyrioides* (Spreng.) Schltr.	1	1

**Table 2:** Descriptive statistics orchid flora of Azerbaijani cemeteries.

Number of cemeteries studied	96
Number of cemeteries hosting orchids	37
Rate of cemeteries hosting orchids	38.5%
Mean (±SD) number of orchid taxa/cemeteries	0.92 (±1.73)
Maximum number of orchid taxa/cemeteries	9
Mean (±SD) number of orchid individuals/cemeteries	44.4 (±173.8)
Maximum number of orchid individuals/cemeteries	1050

**Table 3:** Effect of geographic location on number of orchid taxa per cemetery. Parameter estimates, their standard errors (SE), associated t-values (t) and significance levels (p) are presented.

Full Model					Minimal Model			
	Estimate	SE	t	*p*	Estimate	SE	t	*p*
Intercept	−0.2317	0.2018	−1.148	0.251	−0.1916	0.2017	−0.95	0.342
Altitude	0.0850	0.1746	0.487	0.627				
Latitude	0.5750	0.1943	2.960	0.003	0.5471	0.1968	2.78	0.005
Longitude	0.3305	0.1916	1.725	0.084				

**Table 4:** Effect of geographic location on number of orchid individuals in Azerbaijani cemeteries.

Full Model					Minimal Model			
	Estimate	SE	t	*p*	Estimate	SE	t	*p*
Intercept	3.8439	0.3783	10.161	<0.001	3.8765	0.3788	10.234	<0.001
Altitude	0.0784	0.1801	0.435	0.6635				
Latitude	0.2891	0.1930	1.498	0.1342	0.5770	0.2032	2.839	0.0045
Longitude	0.6063	0.2023	2.997	0.0027				

## Data Availability

All data analyzed in this study are available in Appendix A.

## Appendix A

**Table A1:** Numbering (ID), geographic location, altitude, year of observation and orchid taxa of the 96 cemeteries studied in Azerbaijan. A dash “–” indicates that no orchid taxa were recorded. Generic name abbreviations: *A.—Anacamptis, C.—Cephalanthera, D.—Dactylorhiza, E.—Epipactis, H.—Himantoglossum, L.—Limodorum, O.—Orchis, Op.—Ophrys, S.—Steveniella*.

ID	Settlement	Latitude, Longitude	Alt. (m)	Year	Taxa (Number of Individuals)
01	Şorsulu	39.42429° N, 48.82938° E	26	2018	–
02	TəzəAlvadı	39.09155° N, 48.61027° E	2	2018	–
03	Lənkəran	38.77246° N, 48.83487° E	29	2018	–
04	Lənkəran	38.74200° N, 48.83201° E	19	2018	–
05	Velədi	38.72414°N, 48.82849°E	15	2018	–
06	Şürük	38.69889° N, 48.78815° E	21	2018	–
07	Telman	38.65184° N, 48.80355° E	1	2018	–
08	Kəkülus	38.61265° N, 48.84406° E	17	2018	–
09	Kərgəlan	38.73326° N, 48.79404° E	0	2018	–
10	Shaglakuche	38.72074° N, 48.76931° E	58	2018	–
11	Shaglakuche	38.71618° N, 48.76020° E	60	2018	–
12	Shaglakuche	38.71618° N, 48.74096° E	80	2018	–
13	Shaglakuche	38.71839° N, 48.72004° E	97	2018	–
14	Lerik	38.80257° N, 48.45105° E	803	2018	*O. mascula* (4)
15	Lerik	38.77126° N, 48.41079° E	1108	2018	–
16	Lerik	38.76576° N, 48.42419° E	1062	2018, 2019	*A. papilionacea* (2019: 3), *A. pyramidalis* (2018: 500, 2019: 300), *D. romana* (2019: 20), *Op. caucasica* (2019: 5), *Op. sphegodes* subsp. *mammosa* (2018: 50, 2019: 100), *O. adenocheila* (2018: 5, 2019: 200), *O. mascula* (2019: 5), *O. simia* (2018: 50, 2019: 200), *S. satyrioides* (2019: 1)
17	Ambu	38.75581° N, 48.44571° E	1238	2018	–
18	Gosmalijion	38.69194° N, 48.40264° E	1308	2018	–
19	Laman	38.85799° N, 48.39724° E	800	2018	–
20	Aran	38.87474° N, 48.39668° E	780	2018	–
21	Aran	38.87868° N, 48.39648° E	780	2018	–
22	Bülüdül	38.83971° N, 48.30756° E	805	2018	*A. pyramidalis* (7), *L. abortivum* (4)
23	Züvüç	38.86909° N, 48.30655° E	885	2018	–
24	Yardımlı	38.88330° N, 48.28216° E	1020	2018	–
25	Yardımlı	38.90051° N, 48.25522° E	827	2018	–
26	Yardımlı	38.90921° N, 48.24933° E	720	2018	–
27	Perimbel	38.90218° N, 48.09793° E	1366	2018	–
28	Yardımlı	38.91879° N, 48.31857° E	580	2018	–
29	Yeyənkənd	38.98335° N, 48.57487° E	102	2018	*L. abortivum* (8)
30	Allahyarlı	39.00025° N, 48.60194° E	66	2018	–
31	Göygöl	40.57610° N, 46.30989° E	720	2018	–
32	Zurnabad	40.51304° N, 46.24282° E	958	2018	*A. pyramidalis* (100), *Op. sphegodes* subsp. *mammosa* (8)
33	Dağ Kəsəmən	41.08915° N, 45.38852° E	401	2018	–
34	Poylu	41.15871° N, 45.44410° E	328	2018	–
35	Köçəsgər	41.05043° N, 45.50680° E	456	2018	–
36	Qaraxanlı	41.05042° N, 45.68841° E	346	2018	–
37	Tovuz	40.97800° N, 45.62000° E	440	2018	–
38	İbrahimhacılı	40.89159° N, 45.74460° E	548	2018	–
39	Ağaməmmədli	40.85088° N, 45.73310° E	581	2018	*A. morio* (1)
40	Yanıqlı	40.84016° N, 45.67080° E	669	2018	*A. pyramidalis* (100), *O. simia* (20)
41	Məşədilər	40.87028° N, 45.74312° E	548	2018	–
42	Düyərli	40.91173° N, 45.85246° E	369	2018	–
43	Gəncə	40.71074° N, 46.42177° E	370	2018	–
44	Şiştəpə	40.83121° N, 45.87962° E	592	2018	*A. morio* (8)
45	Şəmkir	40.80390° N, 46.01184° E	559	2018	–
46	Çinarlı	40.78807° N, 46.10889° E	431	2018	–
47	Ağsu	40.53386° N, 48.33778° E	152	2018	*A. pyramidalis* (25), *O. simia* (20)
48	Ağsu	40.56429° N, 48.38220° E	152	2018	–
49	Muğanlı	40.63904° N, 48.50003° E	732	2018	–
50	Muğanlı	40.66912° N, 48.52951° E	878	2018	–
51	Böyük Xınıslı	40.65781° N, 48.61163° E	870	2018	*A. papilionacea* (1)
52	Ağabəyli	40.66747° N, 48.57887° E	927	2018	*A. collina* (1), *A. papilionacea* (465), *A. pyramidalis* (150), *Op. apifera* (1), *Op. sphegodes* subsp. *mammosa* (3), *O. adenocheila* (39), *O. punctulata* (1), *O. simia* (26)
53	Şamaxı	40.64936° N, 48.62496° E	783	2018	–
54	Muğanlı	40.67730° N, 48.55807° E	971	2018	*A. pyramidalis* (9), *Op. apifera* (2)
55	Suraxanı	40.71495° N, 48.47015° E	900	2018	*A. pyramidalis* (1)
56	Kalva	40.72985° N, 48.48152° E	907	2018	*A. pyramidalis* (1)
57	Xatman	40.72947° N, 48.49023° E	862	2018	–
58	Dilman	40.72596° N, 48.49876° E	831	2018	–
59	Məlhəm	40.69225° N, 48.62865° E	1115	2018	*A. papilionacea* (1)
60	Qəleybuğurd	40.75093° N, 48.56726° E	912	2018	*L. abortivum* (10)
61	Əngixaran	40.67143° N, 48.65670° E	978	2018	*A. papilionacea* (2), *A. pyramidalis* (140), *Op. sphegodes* subsp. *mammosa* (8), *Op. oestrifera* (1)
62	Gandov	40.81714° N, 48.31921° E	952	2018	–
63	Müşkəmir	40.81919° N, 48.33202° E	1100	2018	–
64	Lahıc	40.84393° N, 48.37823° E	1214	2018	*A. coriophora* (1), *A. pyramidalis* (7)
65	Qaraqaya	40.79417° N, 48.30885° E	1111	2018	–
66	Talıstan	40.79954° N, 48.20033° E	827	2018	–
67	Talıstan	40.80186° N, 48.20287° E	850	2018	–
68	İkinci Yeniyol	40.75425° N, 48.26252° E	850	2018	*A. pyramidalis* (12), *Op. oestrifera* (1)
69	Təzəkənd	40.73435° N, 48.27164° E	671	2018	*A. papilionacea* (10), *A. pyramidalis* (2)
70	Sabir	40.59207° N, 48.70527° E	567	2018	–
71	Digah	41.38517° N, 48.47876° E	662	2018	*A. pyramidalis* (3)
72	Qirmizi Qəsəbə	41.37358° N, 48.51741° E	607	2018	–
73	Ağbil	41.42650° N, 48.56662° E	410	2018	*C. damasonium* (1), *E*. sp. (2)
74	Zizik	41.38543° N, 48.57021° E	482	2018, 2019	*H. formosum* (2018: 21, 2019: 7), *Op. sphegodes* subsp. *mammosa* (2018: 1)
75	Avadjuk	41.47244° N, 48.39413° E	670	2018	*A. pyramidalis* (1)
76	Hil	41.46756° N, 48.35726° E	770	2018	–
77	Hil	41.46891° N, 48.33767° E	768	2018	-
78	Yasab	41.49661° N, 48.31670° E	787	2018, 2019	*H. formosum* (2018: 4, 2019: 0)
79	Piral	41.50062° N, 48.29514° E	838	2018	*A. pyramidalis* (13), *H. formosum* (2)
80	Hazra	41.50588° N, 48.25472° E	725	2018	*A. pyramidalis* (2)
81	Laza	41.29840° N, 48.11429° E	1703	2018	–
82	Urva	41.40196° N, 48.34058° E	1046	2018	–
83	Qusar	41.41850° N, 48.40676° E	768	2018	*C. rubra* (2)
84	Pirvahid	41.32669° N, 48.65619° E	370	2018	*A. pyramidalis* (6)
85	Tengealti	41.23764° N, 48.62676° E	701	2018	*A. pyramidalis* (10), *C. rubra* (5), *E. turcica* (7), *L. abortivum* (1)
86	Sirt-Chichi	41.22650° N, 48.67541° E	672	2018	–
87	Chichi	41.21673° N, 48.67737° E	538	2018	*A. pyramidalis* (2), *C. rubra* (2), *E. microphylla* (3)
88	Gilanov	41.23790° N, 48.75276° E	325	2018	–
89	Mashrif	41.09305° N, 48.96764° E	420	2018	–
90	Dashli Jalgan	41.08023° N, 48.98348° E	180	2018	*A. collina* (77), *A. papilonacea* (35), *Neotinea tridentata* (3), *Op. sphegodes* subsp. *mammosa* (96), *O. simia* (11)
91	Tıxlı	40.90958° N, 49.10128° E	593	2018	*A. collina* (8), *A*. sp. (6), *Op*. sp. (24)
92	Nugadi	41.31504° N, 48.59641° E	506	2018	*A. pyramidalis* (800), *O. cf. caucasica* (50), *O. simia* (200)
93	Nohurqishlaq	40.95280° N, 47.92485° E	749	2019	*A. coriophora* (5), *A. pyramidalis* (5), *A. morio* (505), *Op. sphegodes* subsp. *mammosa* (10), *Op. oestrifera* (1), *O. ×chabalensis* (*O. punctulata × O*. *stevenii*) (30), *O. punctulata* (10), *O. stevenii* (20)
94	Chukhur Gabala	40.87934° N, 47.69153° E	404	2019	*A. pyramidalis* (5)
95	Şəfili	40.84899° N, 47.69877° E	354	2019	*A. papilionacea* (50), *Op. sphegodes* subsp. *mammosa* (1), *O. punctulata* (3), *O. simia* (15)
96	Gosmalijion	38.67424° N, 48.37322° E	1450	2018	–
